# Digenic mutations of human *OCRL* paralogs in Dent’s disease type 2 associated with Chiari I malformation

**DOI:** 10.1038/hgv.2016.42

**Published:** 2016-12-08

**Authors:** Daniel Duran, Sheng Chih Jin, Tyrone DeSpenza, Carol Nelson-Williams, Andrea G Cogal, Elizabeth W Abrash, Peter C Harris, John C Lieske, Serena JE Shimshak, Shrikant Mane, Kaya Bilguvar, Michael L DiLuna, Murat Günel, Richard P Lifton, Kristopher T Kahle

**Affiliations:** 1 Department of Neurosurgery, Yale School of Medicine, New Haven, CT, USA; 2 Department of Genetics, Yale School of Medicine, New Haven, CT, USA; 3 Howard Hughes Medical Institute, Chevy Chase, MD, USA; 4 Division of Nephrology and Hypertension, Mayo Clinic College of Medicine, Rochester, MN, USA; 5 Department of Biochemistry and Molecular Biology, Mayo Clinic College of Medicine, Rochester, MN, USA; 6 O'Brien Urology Research Center, Mayo Clinic College of Medicine, Rochester, MN, USA; 7 Department of Laboratory Medicine and Pathology, Mayo Clinic College of Medicine, Rochester, MN, USA; 8 Yale Center for Genome Analysis, Yale School of Medicine, Yale University, New Haven, CT, USA; 9 Department of Pediatrics, Yale School of Medicine, New Haven, CT, USA; 10 Department of Cellular & Molecular Physiology, Yale School of Medicine, New Haven, CT, USA

## Abstract

*OCRL1* and its paralog *INPP5B* encode phosphatidylinositol 5-phosphatases that localize to the primary cilium and have roles in ciliogenesis. Mutations in *OCRL1* cause the X-linked Dent disease type 2 (DD2; OMIM# 300555), characterized by low-molecular weight proteinuria, hypercalciuria, and the variable presence of cataracts, glaucoma and intellectual disability without structural brain anomalies. Disease-causing mutations in *INPP5B* have not been described in humans. Here, we report the case of an 11-year-old boy with short stature and an above-average IQ; severe proteinuria, hypercalciuria and osteopenia resulting in a vertebral compression fracture; and Chiari I malformation with cervico-thoracic syringohydromyelia requiring suboccipital decompression. Sequencing revealed a novel, *de novo* DD2-causing 462 bp deletion disrupting exon 3 of *OCRL1* and a maternally inherited, extremely rare (ExAC allele frequency 8.4×10^−6^) damaging missense mutation in *INPP5B* (p.A51V). This mutation substitutes an evolutionarily conserved amino acid in the protein’s critical PH domain. *In silico* analyses of mutation impact predicted by SIFT, PolyPhen2, MetaSVM and CADD algorithms were all highly deleterious. Together, our findings report a novel association of DD2 with Chiari I malformation and syringohydromyelia, and document the effects of digenic mutation of human *OCRL* paralogs. These findings lend genetic support to the hypothesis that impaired ciliogenesis may contribute to the development of Chiari I malformation, and implicates OCRL-dependent PIP_3_ metabolism in this mechanism.

## Introduction

Dent disease is a rare X-linked recessive condition characterized by proximal tubule renal dysfunction leading to hypercalciuria, decreased renal tubular phosphate reabsorption, low-molecular weight proteinuria, aminoaciduria and variable presence of nephrolithiasis, nephrocalcinosis, hematuria and renal failure.^1^ Extrarenal manifestations may also be present, and include intellectual impairment, short stature, growth retardation and rickets in ~30% of patients.^1–3^ Many features of this condition are variable in severity, which led to initial reports of several separate disorders, sharing common characteristics which are collectively referred to as ’X-linked hypercalciuric nephrolithiasis.^4–6^

Advancements in genetic molecular screening methods have provided further insight into the genomic determinants that characterize this disease spectrum. Mutations in *CLCN5* (Xp11.23) encoding a predominantly endosomal-bound Cl^−^/H^+^ exchanger, critically related to endosomal acidification, transepithelial transport and proximal tubule endocytosis, define Dent disease type 1.^7^ To date, ~200 mutations in *CLCN5* have been reported in Dent disease patients,^7^ and account for ~50% of all cases.

Sequencing initiatives in *CLCN5*-negative patients with clinical features of Dent disease led to the discovery of mutations in *OCRL1* (Xq26.1) in this population.^3^ While sharing the renal manifestations present in Dent disease type 1, patients harboring *OCRL1* mutations display a different pattern of extrarenal manifestations, including variable cognitive impairment, subclinical cataracts and umbilical hernias. These phenotypic presentations coupled with *OCRL1* mutations define Dent disease type 2 (DD2; OMIM #300555).^8^

*OCRL1* was named due to the initial association with the Lowe Oculocerebrorenal Syndrome (OCRL; Lowe Syndrome; OMIM # 309000)^7^ which shares several of the phenotypic manifestations of DD2, but is more severe. Notably, there are several clinical differences between Lowe syndrome and DD2. Infants with Lowe syndrome are often born with bilateral congenital cataracts and other eye abnormalities that can impair vision. Approximately 50% of affected patients also develop infantile glaucoma with buphthalmos. Patients exhibit failure to thrive, neonatal hypotonia with resulting motor impairment, seizures and intellectual ability ranging from normal to severe mental retardation.^9^ Renal abnormalities, commonly Fanconi syndrome and hypophosphatemic rickets, are all characteristic of Lowe Syndrome.^9^

*OCRL1* encodes a 5-phosphatase of phosphatidylinositol 4,5-bisphosphate, 1,4,5-trisphosphate and 1,3,4,5-tetrakisphosphate with subcellular localization at the plasma membrane, trans-Golgi complex and primary cilia.^10–12^ This protein has been linked to multiple key intracellular functions including vesicle trafficking, cytoskeleton stability due to α-actin distribution and ciliogenesis, in addition to intrinsic Rho GTPase binding.^12^

*OCRL1* has one known human paralog, *INPP5B*, located on chromosome 1. OCRL1 and INPP5B share homology in the 5-phosphatase, abnormal spindle-like microcephaly associated protein (ASH) and rho GTPase-activating (RhoGAP) domains. The homology diverges in the pleckstrin homology (PH) domain where clathrin-binding sites in *OCRL1* are absent in *INPP5B.*^13^ Knockout of *Ocrl1* in mice (murine ortholog of *OCRL1*) failed to produce a phenotype. However, knockout of both *Ocrl1* and *Inpp5b* resulted in embryonic lethality, suggesting compensatory functions of *Ocrl1* by *Inpp5b.*^14^ Interestingly, an *Ocrl1* and *Inpp5b* null mouse model expressing humanized *INPP5B* rescued the embryonic lethality phenotype and recapitulated the human DD2 phenotype characterized by low-molecular weight proteinuria and aminoaciduria with failure to thrive.^15^ Homozygous knockout of *Inpp5B* resulted in male infertility due to impaired spermatogenesis and decreased sperm mobility, which has been attributed to impaired cilia function.^16^ These studies suggest that while there may be some functional overlap between OCRL1 and INPP5B, the latter may also have distinct functions disparate from those of OCRL1 that have yet to be fully elucidated. To date, no mutations in *INPP5B* have been directly implicated in human disease.

Here, using whole-exome sequencing, we report the first patient with digenic mutations in paralogs *OCRL1* and *INPP5B* resulting in a novel presentation of Dent’s disease type 2 with Chiari I malformation and syringohydromyelia.

## Materials and methods

All procedures in this study comply with Yale University’s Human Investigation Committee and are Human Research Protection Program. Informed consent was obtained from all participants before sample collection.

### Whole-exome sequencing and variant calling

Samples were sequenced at the Yale Center for Genome Analysis following the center's standard protocol. Targeted capture was performed using the Nimblegen SeqxCap EZ MedExome Target Enrichment Kit (Roche Sequencing, Pleasanton, CA, USA) followed by DNA sequencing by 74 base paired-end sequencing on the Illumina HiSeq 2000 instrument as previously described.^17^ Sequence metrics are shown in Supplementary Table 1. Sequence reads were mapped to the reference genome (HG38) with BWA-MEM^18^ and further processed using the GATK Best Practices workflows,^18,19^ which include duplication marking, indel realignment and base quality recalibration. Single nucleotide variants and small indels were called with GATK HaplotypeCaller and annotated using ANNOVAR,^20^ dbSNP (v138), 1000 Genomes (May 2013), NHLBI exome variant server and ExAC (v3).^21^

### Kinship analysis

The relationship between the proband and parents was estimated using the pairwise identify-by-descent calculation in PLINK.^22^

### *De novo* variant detection

*De novo* variants were called using the TrioDenovo program.^23^ TrioDenovo calculates the posterior likelihood of a mutation being a *bona fide de novo* event and assigned a data quality (DQ) score to each variant call. *De novo* candidates were filtered based on the following hard filters: (1) have a minor allele frequency (MAF) ⩽5×10^−3^ in ExAC, (2) pass GATK variant quality score recalibration, (3) have a minimum 10 reads total, 5 alternate allele reads and a 20% alternate allele ratio in proband, (4) have a minimum depth of 10 reference reads and alternate allele ratio <3% in parents (5) are exonic or canonical splice-site variants and (6) have a DQ⩾7 (suggested cutoff by authors of TrioDenovo). Finally, false positives were excluded by *in silico* visualization using Integrative Genomics Viewer (IGV)^24^ and BLAT search.

### Dominant/recessive variant detection

Dominant variants were filtered for rareness (MAF⩽5×10^−5^ across 1000 Genomes, exome variant server or ExAC) and high-quality heterozygotes (pass GATK VQSR, have a minimum eight reads total, have a genotype quality score ⩾20, and have alternate allele ratio ⩾20%). The deleteriousness of missense mutations was predicted by the MetaSVM^25^ rank score (annotated as ’D-Mis’ if MetaSVM score ⩾0.83357). We filtered recessive variants for rare (MAF⩽10^−3^ across 1000 Genomes, exome variant server or ExAC) homozygous and compound heterozygous mutations that exhibited high-quality sequence reads (pass GATK VQSR, have a minimum eight reads total for both proband and parents, and have a genotype quality score ⩾20). Only loss-of-function mutations (nonsense, canonical splice-site and frameshift indels) and D-Mis were considered potentially damaging.

### Targeted *OCRL1* sequencing

The patient was enrolled at the Mayo Clinic’s Rare Kidney Stone Consortium and consent for molecular testing was obtained from the parents. Blood was drawn for DNA isolation and Sanger sequencing was performed on coding exons of *OCRL1* (NM_000276) and flanking intronic regions using M13-tailed primers (Genewiz, South Plainfield, NJ, USA). Primer sequences are available on request. All Sanger chromatograms were analyzed using Mutation Surveyor, version 5.0.1 (Softgenics LLC, State College, PA, USA).

## Results

The patient, an 11-year-old Caucasian male with a history of short stature, proteinuria, hypercalciuria and osteopenia, had undergone an extensive renal evaluation starting at the age of 5 that included an unremarkable renal ultrasound and an unremarkable renal biopsy. He has been treated with a medical regimen that included an angiotensin converting enzyme (ACE) inhibitor and a thiazide diuretic. A diagnosis of Dent’s disease was clinically suspected. He also had a history of occasional headaches and multiple food allergies and exhibited above-average intellect and excelled in school. His father and paternal grandfather have a history of kidney stones. However, his family history is negative for renal, neurological or ophthalmological disease. Notably, his mother is short in stature, with a medical history of ulcerative colitis and interstitial cystitis. She denied any renal symptoms, and did not present with abnormal serum electrolytes, creatinine or proteinuria on previous outpatient evaluation.

**Figure 1:**
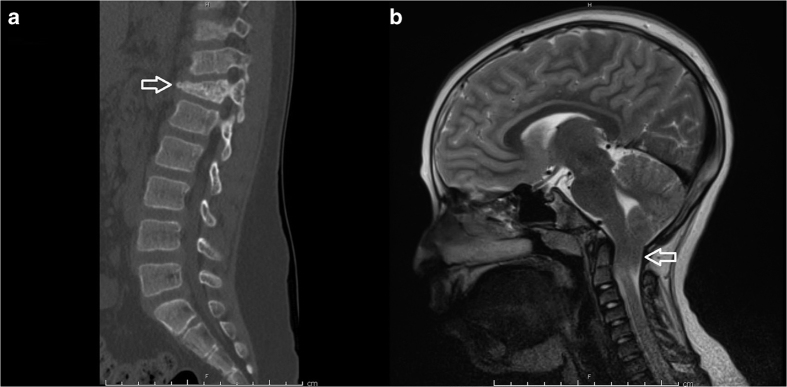
(**a**) Sagittal spine computed tomography image demonstrating a T12 compression fracture (arrow) that occurred after low-impact trauma in the setting of severe osteopenia. (**b**) Sagittal T2-weighted magnetic resonance image demonstrating significant (1.8 cm) herniation of the cerebellar tonsils (arrow) beyond the level of the foramen magnum, hallmark of a Chiari type 1 malformation. This was associated with syringohydromyelia extending through the length of the cervical and thoracic cord, the largest diameter of which measured ~3 mm at the level of C6 and C7.

The neurosurgical consult service was contacted when the subject presented to the emergency room with low back pain 4 days after a minor fall in the sitting position from a height of ~3 feet while playing on his school’s jungle gym. On exam, the patient was normotensive (BP 96/71) and short in stature for his age (height 123 cm, <3rd percentile; weight 34.5 kg 43rd percentile). He was neurologically intact but exhibited point tenderness at his thoracic-lumbar junction. Radiographic evaluation including plain films and a computed tomography of his thoracolumbar spine revealed a T12 compression fracture with ~25% loss of vertebral body height and no significant canal compromise from retroplusion of bony fragments (Figure 1a). Magnetic resonance imaging of the entire spine showed no spinal cord compression from the fracture, but uncovered a significant Chiari I malformation (1.8 cm of tonsillar descent below the level of the foramen magnum; Figure 1b). This was associated with syringohydromyelia extending along the length of the cervical and thoracic cord, the largest diameter measuring ~3 mm at the level of C6 and C7. There was no evidence of hydrocephalus.

Serum labs revealed normal levels of creatinine (0.58 mg/dl), ionized and total serum calcium, and phosphorus. Urine chemistries were remarkable for proteinuria (spot urine=0.86 g/l; protein/creatinine ratio=1.3 mg/mg), hypercalciuria (UCa/Cr ratio=0.38 mg/mg), elevated 1,25 dihydroxy vitamin D (73 pg/ml, normal=25–66 pg/ml), and normal 25 hydroxy vitamin D (37 ng/ml, normal=20–50 ng/ml). Urine β2 microglobulin was markedly elevated (90 mg/l, normal⩽0.23 mg/l). A renal ultrasound was unremarkable without evidence of kidney stones or nephrocalcinosis. A DEXA scan demonstrated severe osteopenia with a Z-score of −2.0. Given these findings, blood samples were collected from the child and his two biological parents. DNAs were isolated and sent for targeted sequencing of the genes known to cause Dent’s disease and whole-exome sequencing. The patient’s lumbar compression fracture was conservatively managed with a thoracolumbosacral orthosis (TLSO) brace. A suboccipital craniotomy with duroplasty and C1 laminectomy were electively performed for the Chairi I malformation months after his compression fracture had successfully healed.

**Figure 2:**
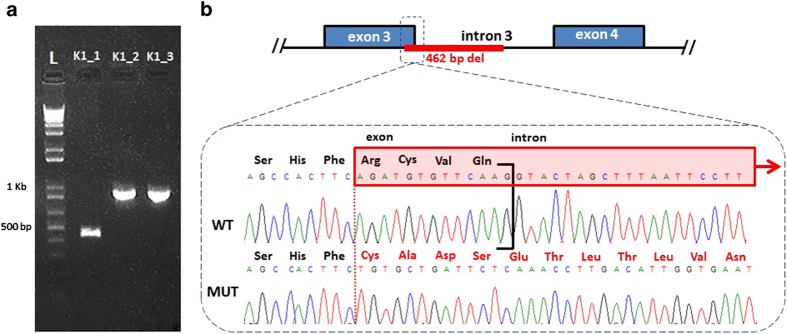
(**a**) Agarose gel electrophoresis of 1 kb Plus DNA ladder and PCR products amplified from the DNAs of affected child [K1_1], mother [K1_2] and father [K1_3]. PCR was performed using primers designed 101 bp 5′ of the Splice Acceptor Site of Exon 3 and 677 bp 3′ of the Splice Donor Site of Exon3 of the *OCRL1* gene. Note the absence of the lower molecular weight fragment in the mother, suggesting *de novo* occurrence of the mutation in the affected child. (**b**) Schematic depicting the mutant sequence, harboring a novel, *de novo* 462 bp deletion: c.187_199+449del (p.Arg63fsX) involving the last 13 nt of exon 3 of *OCRL1.* The mutant sequence shows the frameshift in exon 3 and the out of frame amino acid sequence, assuming the deletion results in inclusion of the residual intron 3.

Screening of the proband demonstrated no mutations in the coding regions of *CLCN5*. Screening of *OCRL1* by PCR and direct Sanger sequencing was performed as described in the Methods section. PCR failed to amplify exon 3 in the child compared with a positive control. Subsequently, PCR employing primers designed 101 bp 5′ of the Splice Acceptor Site of Exon 3 and 677 bp 3′ of the Splice Donor Site of Exon3 of the *OCRL1* gene (available on request) revealed an abnormal fragment ~500 bp smaller when compared with those from both parents, providing evidence of a deletion within the genomic sequence in that region (Figure 2). Absence of the lower molecular weight fragment in the mother suggests this deletion is *de novo*. Targeted Sanger sequencing was then performed to further characterize the lower molecular weight product, which revealed a novel 462 bp deletion removing the final 13 nt of exon 3 and 449 nt of intron 3 (c187_199+449del; Figure 2). This deletion completely removes the exon 3 splice donor site, likely resulting in a fully penetrant splicing defect.

Suspecting the potential contribution of other genetic alterations given this patient’s atypical DD2 phenotype, whole-exome sequencing was performed on genomic DNA from the proband and both parents as described in the Methods section. The sequencing run achieved an average of 98.2 and 95.4% bases with at least 8x and 20x coverage, respectively (Supplementary Table 1). The identify-by-descent shared between the proband and parents is ~50% which confirms the parent–offspring relationship. Variants were called using the GATK Best Practices pipeline^18,19^ and annotated for protein change. The deleterious impact of missense variants was inferred using the MetaSVM algorithm.^25^ False positives were excluded by *in silico* visualization of aligned reads. Mutations that could contribute to significant results were validated by Sanger sequencing.

As parents did not have a history of renal involvement, it was suspected that the disease may be caused by either *de novo* or homozygous mutations in the context of recessive inheritance. To identify *de novo* mutations, the TrioDeNovo^23^ program was used and high-stringency filters were applied as described in the Methods section. We identified two *de novo* mutations in this trio. The first is a tolerated missense mutation in *ZNF644* (p.L442F) and the second is a predicted-deleterious missense mutation in *UBE2D4* (p.D112H; Supplementary Table 2). Both *de novo* mutations are absent among >10^5^ alleles in ExAC. ZNF644, *Zinc-Finger Protein 644*, encodes a zinc-finger transcription factor, interacts with G9a to regulate gene expression during neurogenesis.^26^ In addition, mutations in *ZNF644* have been associated with myopia.^27^ UBE2D4, *Ubiquitin Conjugating Enzyme E2 D4*, encodes a protein that is involved in the p53 and ubiquitination pathways.^28^ However, based on predicted functional impact and biological relevance, *ZNF644* and *UBE2D4* are unlikely to contribute to the phenotype. For the recessive genotypes, we did not identify any genes that harbor homozygotes or compound heterozygotes using an MAF cutoff of 10^−3^.

**Figure 3:**
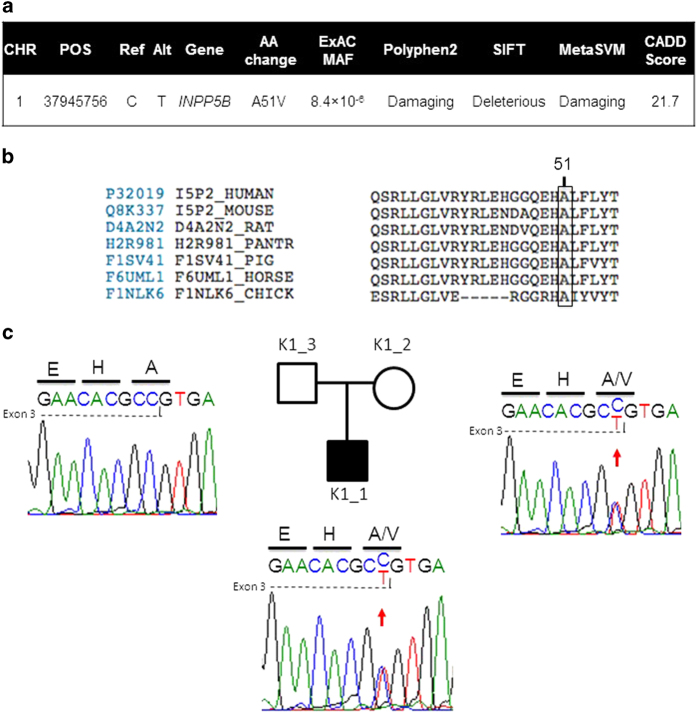
(**a**) Analysis of the INPP5B c.152C>T; p.A51V variant. *In silico* analyses of mutation impact predicted by SIFT, PolyPhen2, MetaSVM and CADD algorithms are present. (**b**) Residue conservation analysis of the p.A51V mutation in INPP5B in orthologous proteins. The INPP5B p.A51V variant is shown with an arrow identifying the corresponding amino acid position. Protein sequences were downloaded from UniProt. The entries used for each species are as follows: P32019 (human), Q8K337 (mouse), D2A2N2 (rat), H2R981 (chimp), F1SV41 (pig), F76UML1 (horse) and F1NLk6 (chicken). (**c**) Family structure and Sanger sequence chromatograms from the trio with the INPP5B p.A51V mutation are shown. Affected individuals are denoted by filled squares while unaffected individuals are denoted by unfilled squares. The DNA sequence and the sequence of the encoded protein are shown in single letter code above sequence traces. Heterozygous mutations were detected in the patient and the mother.

Next, we sought to identify rare (MAF⩽5×10^−5^) transmitted loss-of-function and D-Mis heterozygotes using filters described in the Methods section. In total, we identified 16 (7 loss-of-function and 9 D-Mis; Supplementary Table S3) transmitted protein-altering mutations. Among these, we focused our attention on a maternally inherited missense variant in *INPP5B* (c.152C>T; p.A51V; Figure 3a) in the affected child. The mutation was verified by Sanger sequencing of PCR products in the trio (Figure 3c). This variant is extremely rare, with an overall allele frequency of 8.4×10^−6^, with only 1 allele present in a total of 119,084 alleles in the ExAC database (http://exac.broadinstitute.org/about). Furthermore, no clinical implication of this single reported allele is present in the literature. *INPP5B* c.152C>T; p.A51V is completely conserved across 46 species analyzed (Figure 3b). This residue resides in the PH domain of INPP5B.^13^ *In silico* analyses of the impact of the mutation predicted by SIFT, PolyPhen2, MetaSVM and CADD algorithms were damaging/deleterious (Figure 3a; see Methods section). Nucleotide 152 is the last in exon 3, so we tested if the nucleotide substitution to T was predicted to alter splicing, however, analysis with the BDGP website predicted only a very slight weakening of splicing (0.95–0.94) that is not likely to be pathogenically significant.

## Discussion

We report the first known case of an individual diagnosed with DD2 and Chiari I malformation and found to harbor mutations in the paralogs *OCRL1* and *INPP5B*. Targeted sequencing revealed a novel, *de novo* deletion involving exon 3 of *OCRL1*, and whole-exome sequencing followed by Sanger sequencing revealed a heterozygous, non-synonymous missense variant in *INPP5B.* The latter is extremely rare based on ExAC, changes an evolutionarily conserved residue, resides in the protein’s functional PH domain, and is predicted to be highly deleterious. Mutations in *INPP5B* have not been previously associated with a human disease. In contrast, there have been ~20 cases of DD2 reported in the literature that result from loss-of-function mutations in the 5-phosphatase and PH domains of *OCRL1*. These *OCRL1* mutations result in a spectrum of phenotypes that include low-molecular-weight proteinuria, hypercalciuria, nephrocalcinosis, hyperphosphaturia, aminoaciduria and intellectual disability.^3,29–31^ Structural brain malformations are notably absent in LS and DD2 associated with mutated *OCRL1*. We speculate that the combination of *OCRL1* and *INPP5B* mutations in our patient is responsible for his unique clinical phenotype that includes a significant Chiari I malformation and syringohydromyelia in the cervical and thoracic cord.

Allelic differences in *OCRL1* in Lowe syndrome and DD2 patients may explain the phenotypic variability between these two conditions. The majority of Lowe syndrome patients harbor nonsense or frameshift mutations in exons 9–22 of *OCRL1*, representing the region encoding the protein’s 5-phosphatase activity. Consistent with this finding, functional data demonstrate absence of the *OCRL1* mRNA or a total or near-total lack of 5-phosphatase activity in Lowe syndrome patients.^10,32^ Conversely, DD2 patients harbor missense mutations in this same region of *OCRL1*, yielding protein products with reduced 5-phosphatase activity, or early truncating mutations in exons 1–7.^3^ It is worth noting that early truncating mutations in *OCRL1* often produce clinically mild DD2. This has led some to hypothesize the existence of alternative isoforms of *OCRL1* in the brains and eyes of DD2 patients as a possible explanation for the phenotypic differences between these two conditions.^2^

Furthermore, recent functional studies demonstrate accumulation of phosphatidylinositol 4,5-bisphosphate, abnormal F-actin and α-actin distribution, and impaired ciliogenesis in fibroblasts from both Lowe syndrome and DD2 patients. These changes were more drastic in Lowe syndrome fibroblasts, strengthening the hypothesis that DD2 may represent a milder form of Lowe sydrome.^33^

*In vitro* studies using human and animal cells have shown that the 5-phosphatase and RhoGAP domains are involved with the localization of *ocrl1* to the primary cilium^12,34^ and its ability to modulate the length of the primary cilium.^11^ *INPP5B* has also been shown to be a positive modulator of ciliogenesis and ciliary length.^35^ It has been hypothesized that the essential role of *OCRL1* in ciliogenesis may contribute to the pathology seen in DD2.^33^ The primary cilium is a non-motile projection on most eukaryotic cells, and links changes in the extracellular environment with intracellular signal transduction via a number of signaling pathways, including Sonic Hedgehog and possibly Wnt, key pathways in neurodevelopment.^36,37^ The primary cilium directly determines planar polarity of ependymal cells within the ventricles, and establishes the translational flow of cerebral spinal fluid.^38^ Investigators have speculated DD2 may be a part of a larger family of ’ciliopathies’, which are disorders frequently characterized by renal dysfunction, ataxia, cerebellar malformation and neurological deficits.^39^ Deficits in ciliogenesis and impaired cerebral spinal fluid flow have been associated with hydrocephalus^40–42^ and scoliosis^43^ which are found in increased frequency in patients with Chairi I syndrome.^44^ In the context of a DD2-causing *OCRL1* mutation, we speculate that an *INPP5B* missense mutation may result in impaired ciliogenesis and contribute to Chairi I malformation and syringomyelia.

The genetic basis of Chiari I malformation is not well defined. Genome-wide association studies have identified genes involved in chondrogenesis, such as *EP3000*, *CREBBP*, *SOX9*, *ATF4* and *LHX4.*^45^ A limited number of case reports have presented the association of this condition with abnormal skull morphology, especially due to a small posterior fossa in the context of bone disorders such as X-linked recessive hypophosphatemic rickets (OMIM #300554), or its X-linked dominant counterpart (OMIM # 307800).^46,47^ However, a clear mechanistic association between these conditions has not been accomplished. Further work is needed to define the genetics and mechanism of Chiari I malformation, and to examine further a possible pathogenic role of OCRL-dependent ciliogenesis in its pathogenesis.
